# Galanin plays an important role in cancer invasiveness and is associated with poor prognosis in stage II colorectal cancer

**DOI:** 10.3892/or.2014.3660

**Published:** 2014-12-09

**Authors:** KINUKO NAGAYOSHI, TAKASHI UEKI, KOSUKE TASHIRO, YUSUKE MIZUUCHI, TATSUYA MANABE, HIROMITSU ARAKI, YOSHINAO ODA, SATORU KUHARA, MASAO TANAKA

**Affiliations:** 1 Department of Surgery and Oncology, Graduate School of Medical Sciences, Kyushu University, Higashi-ku, Fukuoka 812-8582, Japan; 2 Department of Bioscience and Biotechnology, Faculty of Agriculture, Kyushu University, Higashi-ku, Fukuoka 812-8581, Japan; 3 Department of Anatomic Pathology, Graduate School of Medical Sciences, Kyushu University, Higashi-ku, Fukuoka 812-8582, Japan

**Keywords:** galanin, colorectal cancer, stage II, prognostic factor

## Abstract

Reliable predictors of tumor recurrence for patients with stage II colorectal cancer (CRC) are needed to select patients who should receive adjuvant chemotherapy. Although galanin (GAL) is expressed in several malignant tumors and is associated with cell proliferation and tumor growth, the prognostic value of *GAL* expression in CRC is poorly understood. We compared *GAL* expression between 56 patients with stage II and III CRC who developed tumor recurrences and 56 patients who did not. The clinical and prognostic significance of *GAL* expression was examined using our data and independent public datasets. We also analyzed the influence of *GAL* expression on the proliferation and invasive activity of CRC cells. Higher expression of *GAL* was associated with tumor recurrence among the CRC patients (P<0.001). Stage II CRC patients who presented with high expression levels of *GAL* had significantly poorer prognosis than those with low expression levels of *GAL* [5-year overall survival: hazard ratio (HR), 7.31; 95% confidence interval (CI), 2.38–24.04; P<0.001; 5-year recurrence-free survival: HR, 3.99; 95% CI, 1.61–9.44; P=0.004], but there was no association between *GAL* expression and survival in stage III CRC patients. These findings were supported by analysis of two public datasets. Functionally, siRNA-mediated silencing of *GAL* resulted in a significant decrease in the proliferative and invasive activities of CRC cells. In conclusion, high expression of *GAL* is associated with poor prognosis of stage II CRC patients and *GAL* expression may be related to the aggressive behavior of CRC.

## Introduction

A significant cause of mortality in patients with colorectal cancer (CRC) is tumor relapse after curative surgical resection. Adjuvant chemotherapy using 5-fluorouracil (5-FU) with or without oxaliplatin increases the survival of patients with stage III CRC by decreasing recurrence (1). However, there is no reliable evidence for a benefit of chemotherapy in patients with stage II CRC. Several clinical and pathologic features have been associated with high risk in patients with stage II CRC, including T4 tumor stage, perforation or obstruction, and poorly differentiated histology (2,3). In patients with stage II CRC treated with adjuvant chemotherapy, their prognosis cannot be accurately predicted and there is no evidence that these patients achieve a benefit from such treatment (4).

The use of molecular markers, such as high-frequency microsatellite instability and chromosomal instability, to indicate the prognosis of stage II CRC patients has been widely investigated. However, due to the heterogeneity of CRC resulting from different molecular features that may develop through multiple pathways (5,6), these molecular characteristics do not reflect all cases of recurrence after curative resection of CRC. Therefore, further stratified studies are needed to investigate other molecular markers that can discriminate individual subsets of CRC patients with poor prognosis who are likely to benefit from chemotherapy.

Galanin (GAL) is a 29 amino acid neuropeptide that is widely distributed in peripheral and central neurons (7). The actions of GAL are mediated through its interaction with at least three specific G-protein-coupled receptor subtypes, namely GalR1, GalR2 and GalR3 (8). Galanin modulates a variety of physiologic processes, including cognition, nociception, memory, feeding, neurotransmitter, hormone secretion and cell proliferation (9–11). In the gastrointestinal tract, GAL plays a role in intestinal contraction (12), regulation of gastric acid secretion, and inhibition of the release of pancreatic peptides (13,14). Although, GAL is expressed in several malignant tumors (11,15–17) and is associated with cell proliferation and tumor growth (11,17–19), the prognostic value of *GAL* expression in cancer patients is not completely understood. Since previous studies have shown that CRC tissue has higher *GAL* expression than normal colonic mucosa (17,20), we speculated that *GAL* expression might play an important role in CRC progression.

In the present study, we conducted a retrospective study to analyze *GAL* expression in stage II CRCs and stage III CRCs and to examine *GAL* expression as an indicator of tumor recurrence of CRC patients. We also investigated the role of *GAL* expression in the proliferative and invasive activities of CRC cells *in vitro*.

## Materials and methods

### Patients and tumor samples

One-hundred and twelve primary tumor samples from 52 patients with stage II CRC and 60 patients with stage III CRC consisting of patients with tumor recurrence and age- and gender-matched patients without recurrence who underwent surgical resection from January 1998 to December 2009 at the Kyushu University Hospital were retrospectively collected, after obtaining each patient’s informed consent for use in research. Twenty-seven normal colonic mucosa samples were also gathered. All samples were frozen in liquid nitrogen immediately after surgical resection and stored at −80°C until RNA extraction. Patients who died in the perioperative period (within 30 days) were excluded. None of the patients received preoperative treatment such as radiation and/or chemotherapy. Of the 112 patients, 79 received postoperative chemotherapy, consisting mainly of 5-FU-based adjuvant therapy, primarily 5-FU + leucovorin (LV) or tegafur-uracil (UFT) + LV, while 33 patients received no treatment. Clinical and pathologic data were obtained from medical records and centrally reviewed for this study. Each tumor was staged according to the American Joint Committee on Cancer TNM staging system and the patients were monitored for tumor recurrence and survival (median follow-up, 61.9 months; range, 9.87–131.6 months). Recurrence was defined as local tumor recurrence, distant metastasis, or peritoneal metastasis. Recurrence was investigated by regular patient checkups as follows: office visits and assays of tumor markers every 3 months for the first 3 years and every 6 months for the next 2 years; colonoscopy every 12 months for the first 3 years; and computed tomography every 6 months for the first 5 years (21). The Kyushu University Hospital Human Research Ethics Committee approved this study.

### RNA extraction and reverse transcription and quantitative real-time PCR

Total RNA was extracted from frozen tumor samples using TRIzol reagent (Invitrogen, Carlsbad, CA, USA). cDNA was synthesized from 500 ng total RNA using the High Capacity cDNA Reverse Transcription kit (Applied Biosystems, Foster City, CA, USA). mRNA expression levels were quantified using quantitative real-time PCR in a 96-well format by a SYBR^®^ Green-based approach using 7500 Fast Real-Time PCR System (Applied Biosystems) and SYBR^®^ Premix Ex Taq™ II (Takara Bio, Inc., Ohtsu, Japan) in a final volume of 20 μl including 100 ng cDNA and 0.4 pmol/μl of each primer. The thermal cycling conditions included an initial denaturation for 30 sec at 95°C and 40 cycles consisting of an annealing step at 95°C for 5 sec and an extension step at 60°C for 34 sec. Each sample was analyzed in triplicate. The sequences of the primers used for PCR are as follows: *GAL* (forward, 5′-CCGGCCAAGGAAAAACGAG-3′ and reverse, 5′-GAGGCCATTCTTGTCGCTGA-3′); *GAPDH* (forward, 5′-CCGGCCAAGGAAAAACGAG-3′ and reverse, 5′-GAGGCCATTCTTGTCGCTGA-3′). The relative expression of *GAL* was calculated by the 2^−ΔΔCt^ method. Data are presented as the relative quantity of target mRNA normalized to expression of *GAPDH* mRNA and relative to a calibrator sample. Each assay was performed three times.

### Cell culture and siRNA transfection

HCT116 cells were obtained from the American Type Culture Collection and DLD-1 cells were provided by the Japan Human Science Foundation. Two individual siRNAs specific for *GAL* (siRNA *GAL1* sense, 5′-CCCUGAACAGCGCGGCUATT-3′ and antisense, 5′-UAGCCCGCGCUGUUCAGGTT-3′; siRNA *GAL2* sense, 5′-GAGCUGCGGCCCGAAGAUGTT-3′ and antisense, 5′-AUCUUCGGCCGCAGCUCCTT-3′) and negative control siRNA were purchased from Sigma-Aldrich (St. Louis, MO, USA). Cells were transfected with siRNA oligonucleotides (20 nmol/l) using Lipofectamine RNAiMAX (Invitrogen) according to the manufacturer’s protocol. *GAL* expression levels were measured 48 h post transfection.

### Matrigel invasion assay and functional separation

The Matrigel invasion assay was performed using the BD Biocoat Matrigel Invasion Chamber according to the manufacturer’s protocol (BD Biosciences, Bedford, MA, USA). Cells (5×10^5^) were seeded in the upper chamber, which was coated with 20 μg/well Matrigel, and cultured for 48 h. Cancer cells that invaded and migrated to the lower surface of the Matrigel-coated membrane were fixed with 70% ethanol, stained with hematoxylin and eosin, and counted in three random fields at ×100 magnification under a light microscope (BZ-9000; Keyence, Osaka, Japan). Results were expressed as the mean number of invading cells. Each experiment was carried out in triplicate wells and independent experiments were repeated. Invasive cells were isolated by functional separation using the Matrigel invasion assay after 72 h in culture (22).

### Cell proliferation assay

Cell proliferation was evaluated by measuring the fluorescence intensity of propidium iodide (PI) as previously described by Zhang *et al* (23). CRC cells were seeded in triplicate in 24-well plates at a density of 2×10^4^ cells/well. After incubation for 24 h, PI (30 μM) and digitonin (600 μM) were added to each well to label nuclei. The fluorescence intensity of PI, corresponding to the total cell number, was measured using an infinite F200 (Tecan; Invitrogen).

### Meta-analysis

We evaluated the prognostic value of *GAL* expression by meta-analysis of two independent public CRC microarray datasets available on the Gene Expression Omnibus in NCBI. We used two independent datasets, GSE14333 (24) and GSE 17538 (25), in which the frozen tissue samples of primary CRCs included stage II CRCs and stage III CRCs, similar to the samples in this study. The expression data were normalized using quantile normalization. We analyzed *GAL* mRNA expression in the datasets and the minimum P-value approach employed in PrognoScan (26) was used to determine the cut-off value for *GAL* expression that optimally divided patients into groups corresponding to high or low expression for microarray data.

### Statistical analysis

Student’s two-way t-test was used to determine statistically significant differences in the average *GAL* expression between CRC and control samples. Clinical and demographic characteristics were analyzed with χ^2^ tests for categorical variables. Survival curves of the patients were conducted with the Kaplan-Meier method and the difference between the curves was compared using the log-rank test. Univariate and multivariate analyses of death and tumor recurrence were performed using Cox’s proportional hazards model. In multivariate analyses, variables included in the final model were selected using a stepwise method to identify significant risk factors for death and tumor recurrence. A probability level of 0.05 was chosen for statistical significance. Statistical analyses were performed with JMP 11.0.2a software (SAS Institute, Cary, NC, USA).

## Results

### Expression of GAL in CRCs and clinicopathological characteristics

**Figure 1:**
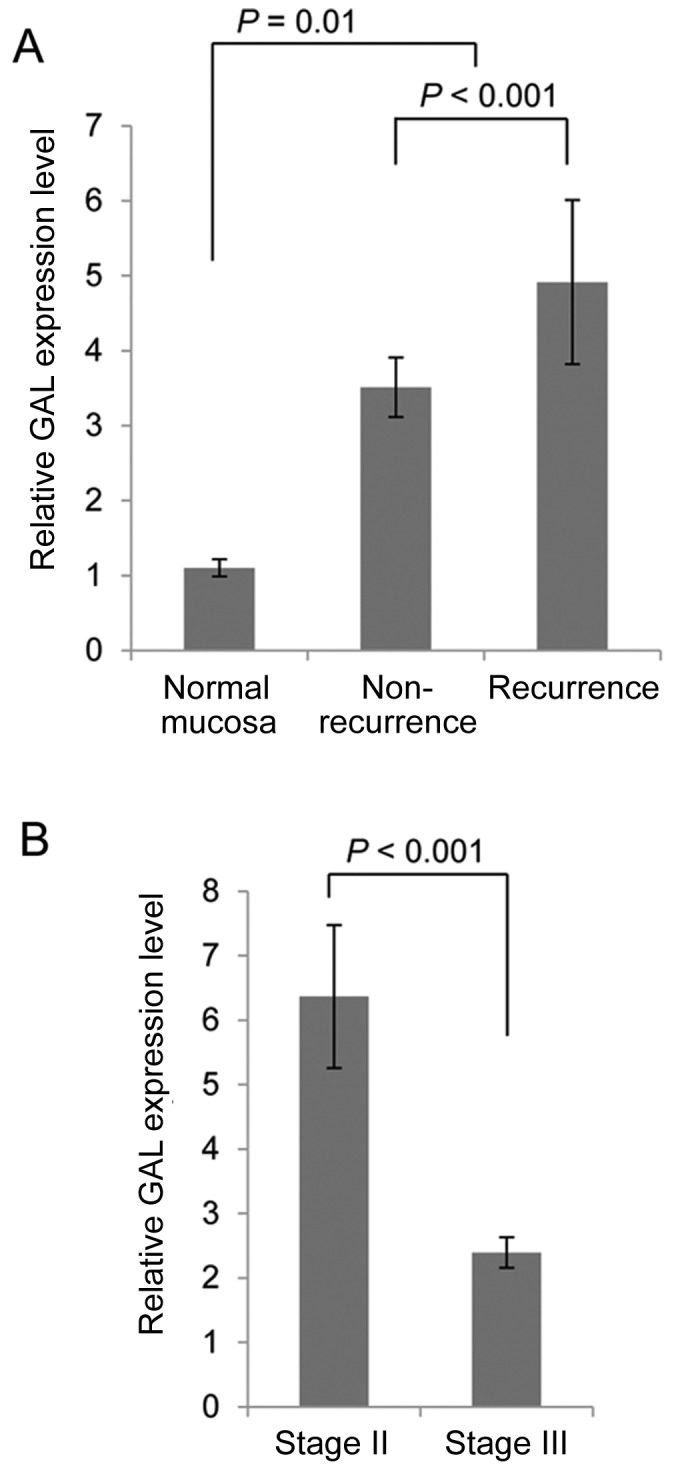
Relative galanin (*GAL*) mRNA expression level by quantitative real-time PCR. (A) Mean *GAL* expression in 112 colorectal cancers (CRCs) (tumors with recurrence and tumors without recurrence) was significantly higher than that in 27 non-cancerous mucosa (P=0.01). Tumors with recurrence showed higher *GAL* expression than those without recurrence (P<0.001). (B) Mean *GAL* expression in stage II CRCs was significantly higher than that in stage III CRCs (P<0.001). Expression was normalized to *GAPDH* mRNA levels.

The *GAL* expression level was significantly higher in 112 CRCs than in 27 non-cancerous mucosa (P=0.01) (Fig. 1A). Quantitative real-time PCR revealed significantly higher expression of *GAL* in CRC with tumor recurrence compared with CRC without tumor recurrence (P<0.001) (Fig 1A) and the *GAL* expression level was higher in stage II CRCs than in stage III CRCs (P<0.001) (Fig. 1B).

We divided 112 CRCs, comprising 52 stage II and 60 stage III CRCs, into *GAL* high and low expression groups using the cut-off value of *GAL* expression level as twice as that of the normal colonic mucosa. The *GAL* high expression group included 11 stage II CRCs (21.2%) and 3 stage III CRCs (5.0%), indicating more stage II CRCs in the *GAL* high expression group than stage III CRCs (P=0.009) (Table I). While there was no significant difference between *GAL* expression and other clinicopathological findings, tumor recurrence occurred more often in the *GAL* high expression group compared with the low expression group, although the difference was not statistically significant (71.4 vs. 46.9%, respectively; P=0.08). Therefore, we further examined the association between the survival of CRC patients and the status of *GAL* expression according to tumor stage.

### High GAL expression is associated with poor prognosis in stage II CRCs but not in stage III CRCs

**Figure 2:**
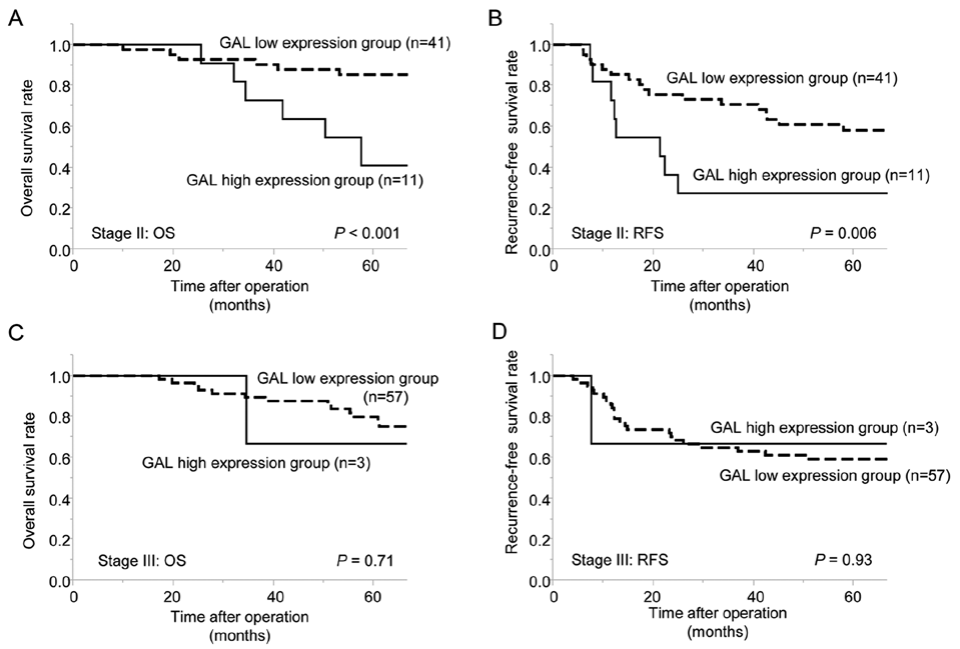
Survival outcomes in colorectal cancer (CRC) patients according to the galanin (*GAL*) expression level. Both 5-year overall survival [(A) 30.3 vs. 82.3%, P<0.001] and 5-year recurrence-free survival [(B) 27.3 vs. 57.5%, P=0.006] were significantly lower in patients with stage II CRC with high *GAL* expression than in those with low *GAL* expression. (C and D) There was no significant difference between *GAL* expression level and survival in patients with stage III CRC.

Stage II CRC patients with high *GAL* expression had a lower 5-year overall survival (5-OS) and 5-year recurrence-free survival (5-RFS) than those with low *GAL* expression (5-OS: 30.3 vs. 82.3%, respectively, P<0.001; 5-RFS: 27.3 vs. 57.5%, P=0.006) (Fig. 2A and B). In patients with stage III CRC, there was no significant difference in 5-OS and 5-RFS according to *GAL* expression (Fig. 2C and D). Univariate analysis revealed that high *GAL* expression was associated with both poor 5-OS and poor 5-RFS in patients with stage II CRC [5-OS: hazard ratio (HR), 5.32; 95% confidence interval (CI), 1.93–14.74; P=0.002; 5-RFS: HR, 2.99; 95% CI, 1.26–6.65; P=0.02] (Table II), while there was no association between *GAL* expression and 5-OS and 5-RFS in patients with stage III CRC. In multivariate analysis, high *GAL* expression was an independent prognostic factor for 5-OS and 5-RFS in patients with stage II CRC (5-OS: HR, 7.31; 95% CI, 2.38–24.04; P<0.001; 5-RFS: HR, 3.99; 95% CI, 1.61–9.44; P=0.004) (Table II), but not in patients with stage III CRC.

### Survival analysis using independent expression profiling of public data

**Figure 3:**
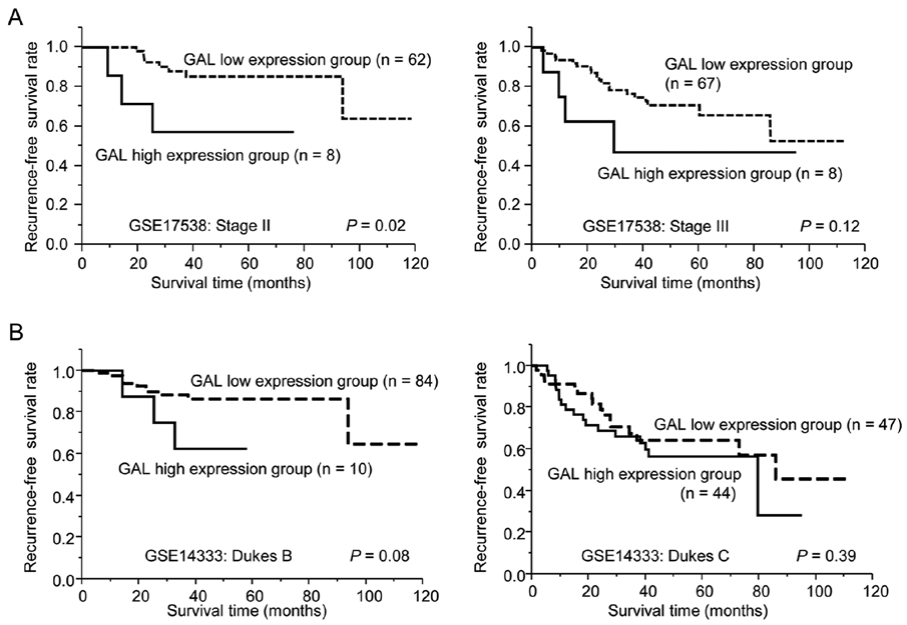
Survival analysis using two independent public expression profiling datasets. Survival analysis of GSE17538 (A) dataset showed that high galanin (*GAL*) expression was significantly correlated with decreased recurrence-free survival in colorectal cancer (CRC) patients without lymph node metastasis (P=0.02), but not in those with lymph node metastasis. Survival analysis of GSE14333 (B) dataset showed that high *GAL* expression tended to be correlated with decreased recurrence-free survival in CRC patients without lymph node metastasis (P=0.08), but not in those with lymph node metastasis.

We confirmed the prognostic value of *GAL* expression using two publicly available independent CRC microarray datasets. In the GSE17538 dataset, stage II CRC patients with high *GAL* expression showed significantly shorter RFS than those with low *GAL* expression. RFS was not significantly different according to *GAL* expression among patients with stage III CRC (P=0.02) (Fig. 3A). Similarly, high *GAL* expression tended to be associated with poor RFS among Dukes’ B patients in the GSE14333 dataset (P=0.08) (Fig. 3B), whereas a significant correlation between high *GAL* expression and poor RFS was shown in Dukes’ A+B patients (P=0.01) (data not shown). High *GAL* expression was not significantly correlated with poor RFS in Dukes’ C patients (Fig. 3B).

### Expression of GAL correlates with the proliferation and the invasive ability of CRC cells

**Figure 4:**
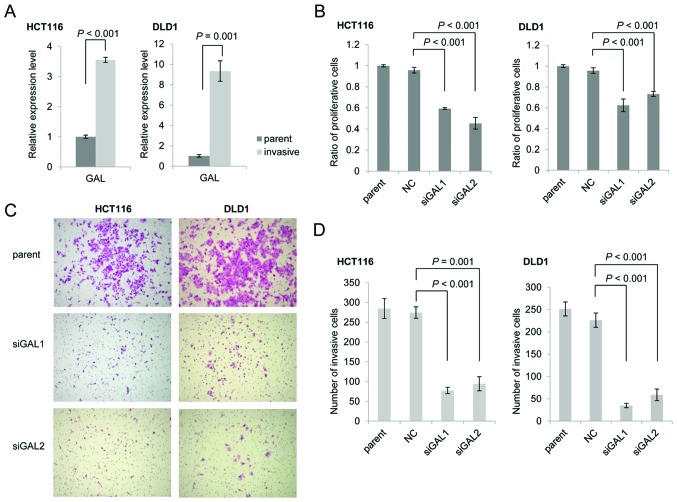
Effect of galanin (*GAL*) silencing on the proliferation and invasion of colon cancer cells. (A) *GAL* expression in invasive colorectal cancer (CRC) cells and parental cells by functional separation. *GAL* expression was significantly higher in invasive CRC cells than parental cells. (B) Cell proliferation ratio in HCT116 and DLD-1 cells. Cell proliferation assay showed a significant decrease in the number of proliferating cells after siRNA-mediated silencing of *GAL* expression. Proliferation is expressed relative to the parent cells. (C) Hematoxylin and eosin staining of invasive HCT116 and DLD-1 cells transfected as indicated after incubation in the assay chambers for 48 h. (D) The invasiveness of HCT116 cells and DLD-1 cells was significantly decreased after silencing of *GAL* expression. Parent, non-transfected cells; NC, negative control siRNA.

To investigate the role of GAL expression in the proliferation and invasion of CRC cells, we examined the proliferation and the invasiveness of two CRC cell lines that express detectable levels of endogenous *GAL* (data not shown). Functional separation based on invasiveness showed that *GAL* expression was significantly higher in invasive CRC cells than in parental cells (HCT116, P<0.001; DLD-1, P=0.001) (Fig. 4A). Suppression of *GAL* expression by siRNA (data not shown) significantly decreased the number of proliferative cells (HCT116, P<0.001; DLD-1, P<0.001) (Fig. 4B). Moreover, suppression of *GAL* expression reduced the number of invasive cells after incubation for 48 h (HCT116, P=0.001; DLD-1, P<0.001) (Fig. 4C and D).

## Discussion

Our data showed that high expression of *GAL* was significantly associated with tumor relapse and poor prognosis of CRC patients. Although *GAL* expression was not associated with conventional clinicopathological risk factors in stage II CRC, such as T4 stage, lymphovascular invasion of tumor cells, and pathologic surgical margin involvement, higher *GAL* expression was an independent poor prognostic factor for OS and RFS in multivariate analysis. These results were verified by analysis of two separate public datasets. Our findings indicate that *GAL* expression might be increased in stage II CRCs that have already developed micrometastases at the time of surgery or those that possess a higher potential for progression and recurrence after resection.

Studies of neuronal cultures from *GAL-*knockout mice demonstrated that GAL and its receptors play a critical developmental role and interact with differentiation factors in a molecular cascade to regulate regeneration and neural cell survival (27,28). Moreover, GAL may function as an autocrine/paracrine modulator to influence tumor cell growth and development in neuroblastoma (29). As described previously (17,20), we found that *GAL* expression was significantly higher in CRCs compared with normal colonic mucosa, especially in CRCs with recurrence. In our preliminary experiment, immunohistochemical analysis showed GAL was expressed in all CRCs examined and localized predominantly to the cytoplasm of the carcinoma cells, whereas none of the non-cancerous colonic mucosa demonstrated positive immunostaining of GAL (data not shown). Together with the decreased proliferative activity of CRC cells after suppression of *GAL* expression, our findings suggest that GAL might act as a direct growth factor. This notion is supported by previous studies describing the mitogenic effect of GAL through the MAP kinase pathway (30,31). Moreover, we showed that *GAL* expression was higher in invasive cells than in corresponding parental cells and that silencing of *GAL* expression significantly decreased the invasive activity of CRC cells. Thus, the subpopulation of CRC cells that sustain high *GAL* expression may be more aggressive and have the potential to cause tumor recurrence in CRC patients.

Contrary to the results of this study, high expression of GAL receptors induces antiproliferative effects by inducing apoptosis (19,32) and stimulation of *GALR1*-overexpressing oral squamous carcinoma cells with exogenous *GAL* induces *ERK* activation and is associated with suppression of cell proliferation and tumor growth *in vivo* (33). However, Stevenson *et al* (34) showed that *GALR1/GAL* silencing downregulates *FLIP_L_* and activates caspase-8-dependent apoptosis in CRC cells, and thus suggested that high *GAL* expression would promote high *FLIP* expression and result in a more aggressive phenotype and chemotherapy resistance. Although the molecular dynamics of *GAL* expression are currently unknown due to the lack of detailed functional data on tumorigenesis and inconsistent data regarding the impact of GAL-GALR signaling on the proliferative activity of tumor cells, several previous reports and our data support the notion of an oncogenic effect of *GAL* in CRC development. Silencing of *GAL* promoted an antiproliferative effect and decreased the invasive activity of CRC cells, suggesting that GAL-GALR signaling might also be a therapeutic target for CRC. As Kim *et al* (17) showed that GAL levels in the serum of CRC patients were significantly higher than those found in normal subjects, the overexpression of GAL in CRCs leads us to propose GAL as a potential marker for CRC screening.

The association between high *GAL* expression and worse prognosis was not observed in stage III CRC. Microarray analysis has shown significantly different expression profiles of many genes between lymph node-positive and -negative tumors, and pathways of immune surveillance, cell motility, and apoptosis might be differentially regulated between stage II and III CRC (35). Thus, the significance of *GAL* expression for tumor proliferation and invasion may differ according to stage. The mechanism of recurrence is proposed to involve the dissemination of cancer stem cells that are characterized by pluripotency and are capable of propagating into metastases at distant sites (36). Because *GAL* is considered to be a marker of multipotent stem cells (37,38), the significant correlation between high *GAL* expression and tumor metastasis, together with the aggressive behavior of CRC cells with high *GAL* expression, indicate a potential role of *GAL* in the dissemination of cancer stem cells in stage II CRC.

Our study has some limitations. The number of patients was too small to draw firm conclusions and additional analysis in a larger patient cohort is required. Additionally, the autocrine and paracrine signal network regulating GAL and its receptors should be investigated to further understand the role of GAL expression in CRC development.

In conclusion, our results showed that high expression of *GAL* is associated with poor prognosis in stage II CRC patients and suggest that *GAL* plays a significant role in the invasion and proliferation of CRC cells. Although further large studies are required, our findings indicate the possibility that GAL-GALR signaling may serve as a prognostic marker and a therapeutic target in patients with stage II CRC.

## Figures and Tables

**Table I:** Association between the clinicopathological characteristics of the 112 colorectal cancer patients and GAL expression.

	GAL expression		
		
Variables	High (n=14)	Low (n=98)	P-value
Age (years)			
≥65	8 (57.1)	38 (38.8)	
<65	6 (42.9)	60 (61.2)	0.20
Gender			
Male	9 (64.3)	49 (50.0)	
Female	5 (35.7)	49 (50.0)	0.31
Location			
Colon	7 (50.0)	65 (63.3)	
Rectum	7 (50.0)	33 (33.7)	0.24
pT stage			
T2	0 (0.0)	8 (8.2)	
T3	12 (85.7)	81 (82.6)	
T4	2 (14.3)	9 (9.2)	0.30
Stage			
II	11 (78.6)	41 (41.8)	
III	3 (21.4)	57 (58.2)	0.009^a^
Histology			
Diff	11 (78.6)	85 (86.7)	
Undiff	3 (21.4)	13 (13.3)	0.44
Lymphatic invasion			
Negative	10 (71.4)	59 (60.2)	
Positive	4 (28.6)	39 (39.8)	0.41
Venous invasion			
Negative	10 (71.4)	52 (53.1)	
Positive	4 (28.6)	46 (46.9)	0.19
Histological surgical margin			
Negative	14 (100.0)	94 (95.9)	
Positive	0 (0.0)	4 (4.1)	0.30
Adjuvant chemotherapy			
Done	9 (64.3)	70 (71.4)	
Not done	5 (35.7)	28 (28.6)	0.59
Tumor recurrence			
Negative	4 (28.6)	52 (53.1)	
Positive	10 (71.4)	46 (46.9)	0.08

a Statistical significance (P<0.05).

Diff, differentiated tumor; Undiff, undifferentiated tumor; GAL, galanin.

**Table II:** Hazard ratios of 52 stage II and 60 stage III colorectal cancer patients.

			5-OS				5-RFS			
				
			Univariate analysis		Multivariate analysis		Univariate analysis		Multivariate analysis	
						
Stage	Variable		HR (95% CI)	P-value	HR (95% CI)	P-value	HR (95% CI)	P-value	HR (95% CI)	P-value
II	Age (years)	≥65/<65	1.03 (0.37–2.77)	0.95			1.05 (0.47–2.28)	0.89		
	Gender	Female/male	1.39 (0.51–3.93)	0.51			1.32 (0.61–2.91)	0.48		
	Location	Rectum/colon	1.28 (0.43–3.51)	0.64			1.29 (0.57–2.80)	0.53		
	pT stage	T3/T4	2.44 (0.38–9.12)	0.30	5.60 (0.80–25.20)	0.08	1.10 (0.18–3.74)	0.90	1.93 (0.30–7.23)	0.43
	Histology of tumor	Undiff/diff	3.16 (0.72–9.96)	0.12			1.45 (0.34–4.18)	0.56		
	Lymphatic invasion	Positive/negative	1.42 (0.50–3.83)	0.49			0.81 (0.33–1.81)	0.62		
	Venous invasion	Positive/negative	1.48 (0.54–4.01)	0.44	1.11 (0.34–3.40)	0.85	1.44 (0.66–3.16)	0.35	1.78 (0.75–4.17)	0.19
	Histological surgical margin	Positive/negative	63.15 (5.90–1,382.45)	0.002^a^	205.63 (14.34–5,824.67)	<0.001^a^	5.18 (0.81–18.91)	0.08	5.85 (0.83–26.11)	0.07
	Adjuvant chemotherapy	Not done/done	2.69 (0.99–7.60)	0.052	2.13 (0.73–6.45)	0.16	1.73 (0.77–3.87)	0.18	1.61 (0.69–3.75)	0.27
	*GAL* expression	High/low	5.32 (1.93–14.74)	0.002^a^	7.31 (2.38–24.04)	<0.001^a^	2.99 (1.26–6.65)	0.02^a^	3.99 (1.61–9.44)	0.004^a^
III	Age (years)	≥65/<65	2.21 (0.81–6.13)	0.12			1.06 (0.47–2.27)	0.89		
	Gender	Female/male	1.54 (0.57–4.32)	0.39			1.17 (0.55–2.52)	0.68		
	Location	Rectum/colon	1.28 (0.43–3.51)	0.64			1.48 (0.67–3.16)	0.33		
	pT stage	T3/T2	2.12 (0.41–38.80)	0.42			1.07 (0.36–4.57)	0.91		
		T4/T2	9.30 (1.34–183.96)	0.02^a^	2.97 (0.73–10.81)	0.12	2.93 (0.71–14.37)	0.13	1.90 (0.60–4.98)	0.25
	pN stage	N2/N1	3.59 (1.31–10.74)	0.01^a^	3.55 (1.22–11.26)	0.02^a^	2.23 (1.03–4.78)	0.04^a^	2.14 (0.95–4.76)	0.07
	Histology of tumor	Undiff/diff	1.48 (0.54–4.01)	0.44			2.40 (0.93–5.52)	0.07		
	Lymphatic invasion	Positive/negative	0.44 (0.14–1.24)	0.12	0.53 (0.15–1.67)	0.28	0.84 (0.38–1.80)	0.65		
	Venous invasion	Positive/negative	2.22 (0.82–6.52)	0.12			3.27 (1.49–7.67)	0.003^a^	3.14 (1.40–7.49)	0.005^a^
	Histological surgical margin	Positive/negative	5.26E-09 (3.44–3.44)	0.28			4.04 (0.63–14.53)	0.12		
	Adjuvant chemotherapy	Not done/done	1.21 (0.39–5.35)	0.76	2.03 (0.60–9.53)	0.27	1.31 (0.53–3.93)	0.58	2.33 (0.86–7.77)	0.1
	*GAL* expression	High/low	1.47 (0.08–7.34)	0.73			0.91 (0.05–4.33)	0.93		

a Statistical significance (P<0.05).

CI, confidence interval; HR, hazard ratio; diff, differentiated tumor; undiff, undifferentiated tumor; 5-OS, 5-year overall survival; 5-RFS, 5-year recurrence-free survival.
